# Quantitative estimation of pleural effusion volume on chest radiography: comparison of radiologists and a general-purpose multimodal AI model using CT as the reference standard

**DOI:** 10.1186/s41747-026-00790-3

**Published:** 2026-09-10

**Authors:** Dominik Schiller, Jakub Pristoupil, Pavlina Polaskova, Martina Sterclova, Lukas Lambert

**Affiliations:** 1 Department of Medical Imaging, Motol and Homolka University Hospital and Second Faculty of Medicine, Charles University, Prague, Czech Republic; 2 Department of Pneumology, Motol and Homolka University Hospital and Second Faculty of Medicine, Charles University, Prague, Czech Republic

**Keywords:** Artificial intelligence, Chest radiography, CT volumetry, Pleural effusion, Quantitative imaging

## Abstract

**Objective:**

Chest radiography provides limited precision of pleural effusion volume estimates. This study compares volume estimates from experienced radiologists and a multimodal artificial intelligence (AI) model, using computed tomography (CT)-derived volumetry as the reference standard.

**Materials and methods:**

In this retrospective observational study, same-day chest CT and radiographs from adult patients with CT-confirmed pleural effusion (≥ 20 mL) were evaluated. Manually segmented pleural effusion volumes from CT served as the reference standard. Two board-certified radiologists independently estimated left and right pleural fluid volume on radiographs. A general-purpose multimodal AI model analyzed each radiograph using a standardized prompt and generated volume estimates for each hemithorax.

**Results:**

Eighty-eight patients (72.0 ± 14.4 years (mean ± standard deviation); 56% male) were included, and 176 hemithoraces were analyzed. Median CT-derived pleural effusion volume was 389 mL (interquartile range 188–703 mL). Correlation with CT volumes was moderate for both radiologists (intraclass correlation coefficient 0.64 and 0.74) and the AI model (0.66). Mean absolute error was 300 mL (95% confidence interval 252 to 356 mL) for radiologist #1, 250 mL (216 to 285 mL) for radiologist #2, and 264 mL (227 to 299 mL) for AI. For detection of effusions ≥ 300 mL, AUC values were 0.84 (radiologist #1), 0.90 (radiologist #2), and 0.85 (AI).

**Conclusion:**

Quantitative estimation of pleural effusion volume on chest radiography demonstrates only moderate agreement with CT, regardless of whether the interpretation is performed by experienced radiologists or a general multimodal AI model. This limitation appears intrinsic to the imaging modality.

**Key Points:**

***Question*** Can a general-purpose multimodal AI model estimate pleural effusion volume on chest radiographs with accuracy comparable to experienced radiologists, using CT volumetry as reference?

***Findings*** Across 88 patients (176 hemithoraces), radiologists and AI showed comparable moderate agreement with CT (mean absolute errors 250–300 mL; intraclass correlation coefficient 0.64–0.74); chest radiography is intrinsically imprecise.

***Relevance statement*** Chest radiography supports only coarse pleural effusion volume estimation regardless of interpreter; however, a general-purpose multimodal AI model achieved accuracy comparable to that of experienced radiologists.

**Graphical Abstract:**

**Figure:**
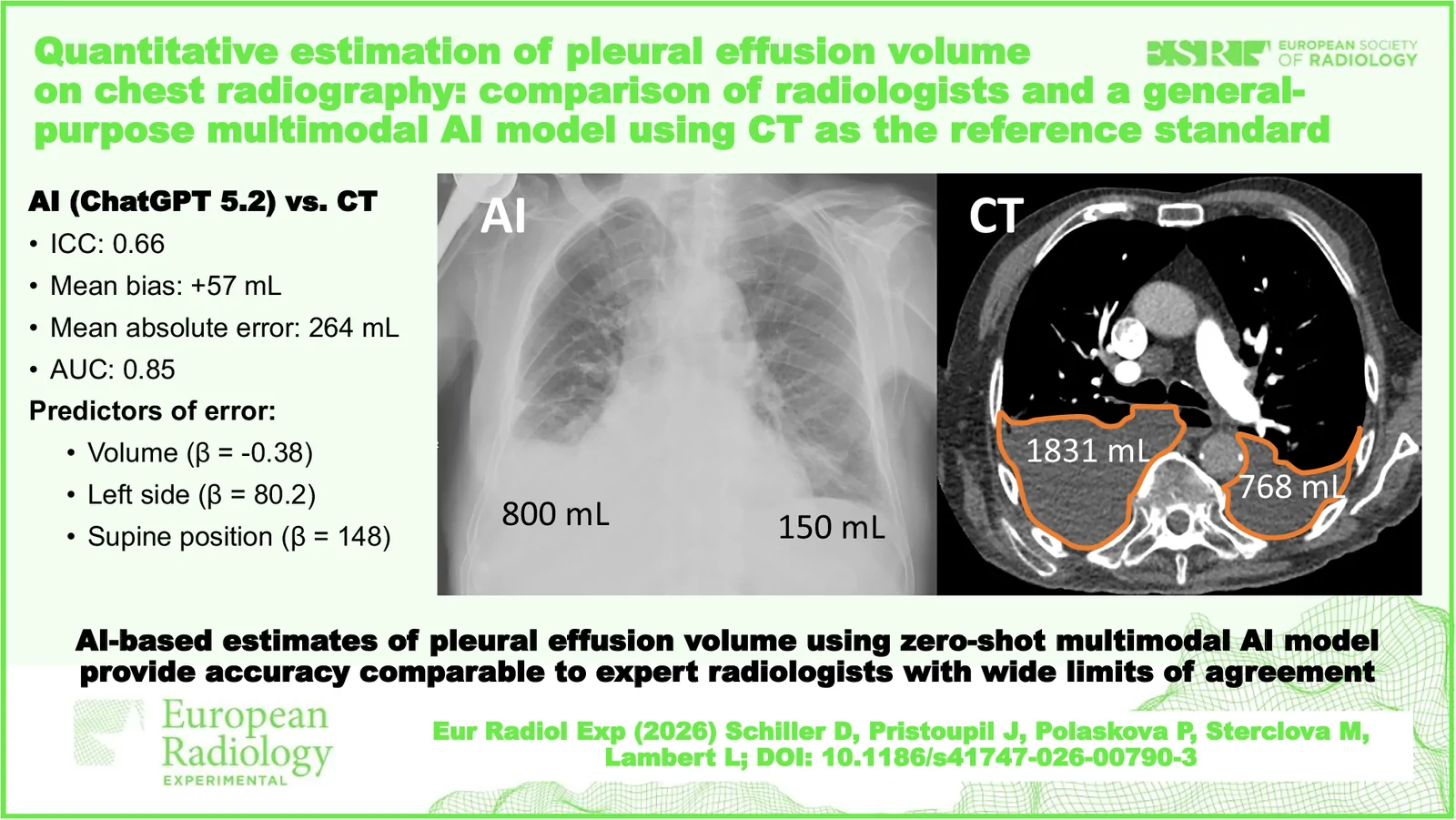

## Background

Pleural effusion is a common radiological and clinical finding associated with a broad range of disorders. Imaging plays a central role in its detection and management, with chest radiography remaining the most widely used first-line modality because of its availability, speed, and low radiation dose [1]. However, classic radiographic signs provide only indirect information about fluid volume, and their visibility depends strongly on patient positioning and technical factors [2, 3]. Quantitative estimation of pleural effusion volume on chest radiographs is a challenging task that requires integrating multiple radiographic cues and implicit geometric inference about fluid distribution within the thoracic cavity [2]. Quantitative estimation, even if approximate, may provide clinically relevant information for early decision-making and standardized communication before confirmatory imaging is performed.

Artificial intelligence (AI) has recently shown promising results in automated analysis of chest radiographs [4, 5]. Deep learning models trained specifically for thoracic imaging tasks have demonstrated high performance in detecting pleural effusion and other thoracic abnormalities [5]. However, most AI research has focused on binary detection tasks, and quantitative estimation of pleural effusion volume from chest radiographs remains largely unexplored. In addition, most studies rely on radiologist annotations as ground truth rather than objective volumetric measurements [6, 7]. While convolutional neural networks are optimized for task-specific detection and segmentation, multimodal generative models combine visual input with implicit reasoning and prior knowledge. Estimating pleural effusion volume from radiographs requires geometric inference from projections, making it a suitable test case for such models.

The aim of this study was to compare pleural effusion volume estimates from chest radiographs obtained by experienced radiologists and by a multimodal AI model using zero-shot prompting, with CT-derived volumetry as the reference standard. We hypothesized that AI would achieve accuracy comparable to that of experienced radiologists, while both approaches would show only moderate agreement with CT due to inherent limitations of chest radiography. Unlike prior studies that primarily focus on binary detection or use radiologist annotations as reference standards, this study directly compares quantitative pleural effusion volume estimates from radiographs across radiologists and a multimodal AI model, using CT-derived volumetry as an objective reference standard.

## Materials and methods

This observational study was conducted in accordance with the principles of the Declaration of Helsinki. Ethical approval was obtained from the Ethics Committee of Motol and Homolka University Hospital (EK-17/23, 11/2/2026). The requirement for informed consent was waived due to the retrospective nature of the study and the use of anonymized imaging and clinical data.

### Study population and inclusion criteria

Consecutive patients were identified through a retrospective search of the institutional radiology information system for adult individuals who underwent both computed tomography (CT) and chest radiography on the same calendar day between January 1, 2025 and December 31, 2025, and the CT report included expressions indicating the presence of pleural effusion and its synonyms.

Eligible examinations were included if the following criteria were met: (1) CT examination covering the entire thorax (≤ 5 mm slice thickness and increment), allowing reliable quantification of pleural effusion; (2) a corresponding chest radiograph performed on the same day.

The following examinations were excluded: (1) incomplete thoracic CT coverage; (2) absence of pleural effusion measuring at least 20 mL on CT in at least one hemithorax; (3) pleural effusion drainage between chest x-ray and CT; (4) insufficient technical quality of radiographs; (5) unavailable data.

This selection strategy resulted in enrichment of positive cases and consequently introduced a spectrum bias. Consequently, the study population does not reflect the full clinical spectrum, particularly very small or absent effusions, which may limit generalizability and affect diagnostic performance metrics, including ROC analysis. This design ensured a sufficiently broad distribution of pleural effusion volumes, which is necessary for meaningful assessment of quantitative estimation error and comparison between methods. Pleural effusions smaller than 20 mL on CT were not considered, as this threshold was regarded as the minimum volume for reliable detection and segmentation.

### CT evaluation

Chest CT served as the reference standard for quantifying pleural effusion. CT examinations were performed on four facility CT scanners (Aquilion ONE, Aquilion PRIZM, and Aquilion PRIME, Canon Medical Systems; Somatom Force, Siemens Healthineers) and reviewed using Syngo Via client workstation (Siemens Healthineers). Volumetric assessment of pleural fluid was based on manual segmentation. The segmentation was performed by a radiology resident using contouring with extrapolation between slices. Axial images with a slice thickness of 3 mm were used uniformly across all scanners, as this represents a pragmatic balance between reducing partial volume effects and maintaining stable, clinically representative image quality. For each hemithorax, the volume was recorded in milliliters and used as the reference standard. The measurement of pleural fluid volume was repeated in 20 randomly selected patients by the resident after a washout period of at least 4 weeks and also by a board-certified radiologist.

### Chest radiograph evaluation

Chest radiographs were independently evaluated by two board-certified radiologists experienced in thoracic imaging (radiologist R1, 19 years’ experience; radiologist R2, 9 years’ experience), who were blinded to CT images and measurements and from each other.

For each radiograph, the readers performed the following evaluations: (1) estimation of pleural effusion volume in milliliters for each hemithorax; and (2) assignment of a confidence score reflecting the perceived reliability of the estimate on a 5-point Likert scale (1—very low; 2—low; 3—moderate; 4—high; 5—very high).

Volume estimates were based on established radiographic signs of pleural effusion, including costophrenic angle blunting, meniscus appearance, layering of pleural fluid, and mediastinal displacement patterns. Each radiologist repeated the estimation of pleural fluid volume in 20 randomly selected patients after a washout period of at least 4 weeks.

Patient positioning was estimated from the radiology information system and verified against image features and DICOM (Digital Imaging and Communications in Medicine) headers (standing, sitting, or supine).

### AI-based radiograph evaluation

Following a prescreening of several widely accessible large language models between 17th and 23rd January 2026 including ChatGPT 5.2 (OpenAI), Grok 4.1 (xAI), Microsoft Copilot (GPT 5) (Microsoft), Claude Sonnet 4.5 (Anthropic), and Gemini 3 Flash (Google) for their appropriateness for the assessment of pleural effusion on chest radiographs, ChatGPT with current version 5.2 was chosen as the best performing. Model prescreening was performed independently of the study dataset and based on qualitative assessment, minimizing the risk of data leakage.

Chest radiographs were analyzed using a commercial version of ChatGPT 5.2 between 12^th^ and 25^th^ February 2026. The AI model was accessed via a browser window (Google Chrome v. 144). Default settings were used; no additional parameter adjustments (including temperature) were made. A custom prompt with code shown below required only image input. Each image was transferred to the AI interface using a standard Windows screen capture tool and inserted into the prompt.

The AI system was required to generate: (1) estimated pleural effusion volume in milliliters for each hemithorax; and (2) a confidence score on a 5-point Likert scale.

To evaluate the AI model's within-session output consistency, each radiograph was analyzed four times within a single chat session. Because all four outputs were produced in a single response, this reflects within-session consistency rather than independent test-retest reliability. To address this concern, 20 randomly selected radiographs were additionally re-evaluated in independent sessions using the same version of ChatGPT on 25^th^ April 2026.

The prompt was structured as follows:

**Table:** 

“You are a medical imaging analysis model with expertise in thoracic radiology. Your task is to analyze the provided chest X-ray image and generate a structured radiological assessment focused on quantifying pleural fluid. Your analysis must be grounded in established radiological principles, geometric estimation methods, and known limitations of plain radiography.
1. Pleural fluid assessment
Report a best estimate of pleural fluid volume in milliliters on each side.
If no pleural fluid is detectable, report the estimated volume as 0 mL.
Provide your confidence on a 5-point Likert scale (1—very low; 2—low; 3—moderate; 4—high; 5—very high)
Output Format
Present your results in the following structured format:
Right pleural space: < estimated volume in mL > , Confidence: < 1–5 >
Left pleural space: < estimated volume in mL > , Confidence: < 1–5 >
Repeat the analysis three more times”

### Statistical analysis

All statistical analyses were performed using R statistical software (version 4.2.3; R Foundation for Statistical Computing) and GraphPad Prism (GraphPad Software). Data distribution was assessed using the D’Agostino–Pearson omnibus test and visual inspection of histograms and Q–Q plots. Continuous variables are reported as mean ± standard deviation for normally distributed data and as median with interquartile range (IQR) for non-normally distributed data. Outliers were assessed using Grubbs’ test (*α* = 0.05). No observations were excluded from the primary analysis. The impact of identified outliers was examined in a secondary sensitivity analysis. The unit of analysis was the hemithorax. Because the two hemithoraces from a single patient are not statistically independent, clustering was applied at the patient level where appropriate.

Agreement between CT-derived pleural effusion volumes and estimates obtained from chest radiographs by radiologists and the AI model was evaluated using the intraclass correlation coefficient (ICC), absolute error (AE), and Bland–Altman analysis. Calibration plots were generated as supplementary analyses.

Interobserver agreement between the two radiologists, as well as the repeatability of both the AI-derived and the radiologists’ assessments, was evaluated using the ICC derived from a two-way mixed-effects model for absolute agreement.

For comparisons of estimation error between methods, forest plots were generated to visualize between-method differences with 95% confidence intervals.

An exploratory receiver operating characteristic (ROC) analysis was performed to evaluate the ability of radiologists and AI estimates to detect clinically significant pleural effusion (defined as CT-derived volume ≥ 300 mL) [8]. The optimal threshold was determined using the Youden index. Area under the ROC curve (AUC) values were compared using the DeLong test.

Exploratory linear regression models were constructed to identify patient- and acquisition-related predictors of estimation error relative to CT.

Sample size estimation was performed using a paired-design power calculation, assuming an error of 100 mL, a standard deviation of paired differences of 200 mL, a significance level of 0.05, and 80% statistical power. The minimum required sample size was calculated to be 32 paired observations.

A two-sided *p*-value < 0.05 was considered statistically significant.

## Results

**Fig. 1:**
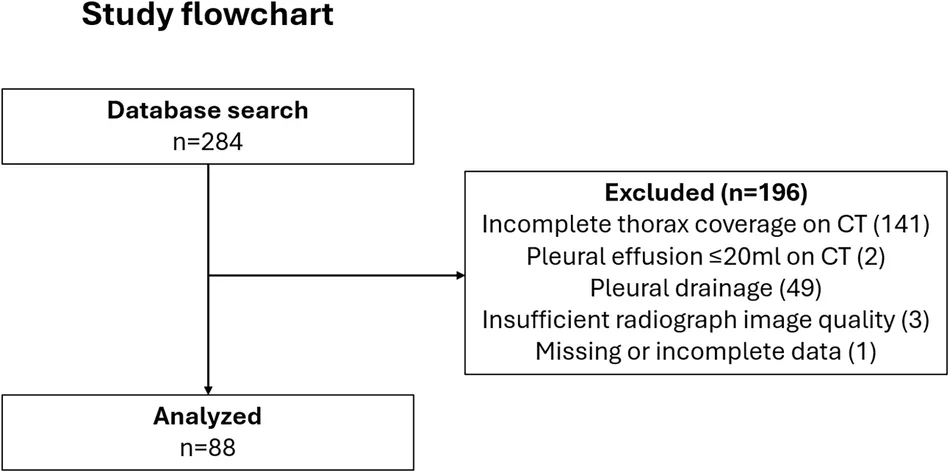
Study flowchart

Between 1st January and 31st December 2025, a total of 284 adult patients who underwent both chest CT and chest radiography on the same day, with pleural effusion reported, were identified. After applying the exclusion criteria, 88 examinations remained, and 176 hemithoraces were analyzed (Fig. 1).

The mean age of the study population was 72.0 ± 14.4 years, with 49 men (56%) and 39 women (44%). The median body mass index was 24.8 kg/m² (IQR 22.1–29.3). The median time interval between chest radiography and CT was 3.0 h (IQR 1.50–5.19 h). Chest radiographs were acquired in standing position in 41% of cases, supine position in 39%, and sitting position in 20%.

**Fig. 2:**
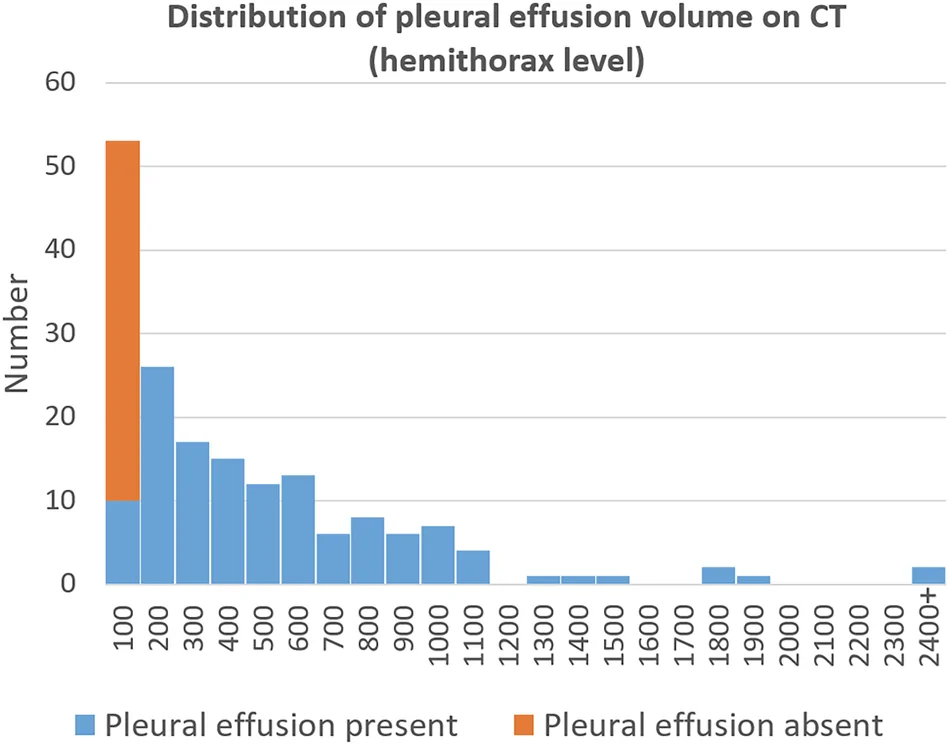
Distribution of pleural fluid volume for the left and right sides. The orange bar represents an absent pleural effusion

Pleural effusion was unilateral in 43 (49%) patients. In pleural spaces with pleural effusions its median volume was 389 mL (IQR 188–703 mL, Fig. 2). The most common primary etiologies of pleural effusion were heart failure (44%), pneumonia (17%), postoperative status (16%), and malignancy (11%), with less frequent causes including post-traumatic and reactive effusions (3% each), and other or uncertain causes (5%).

**Fig. 3:**
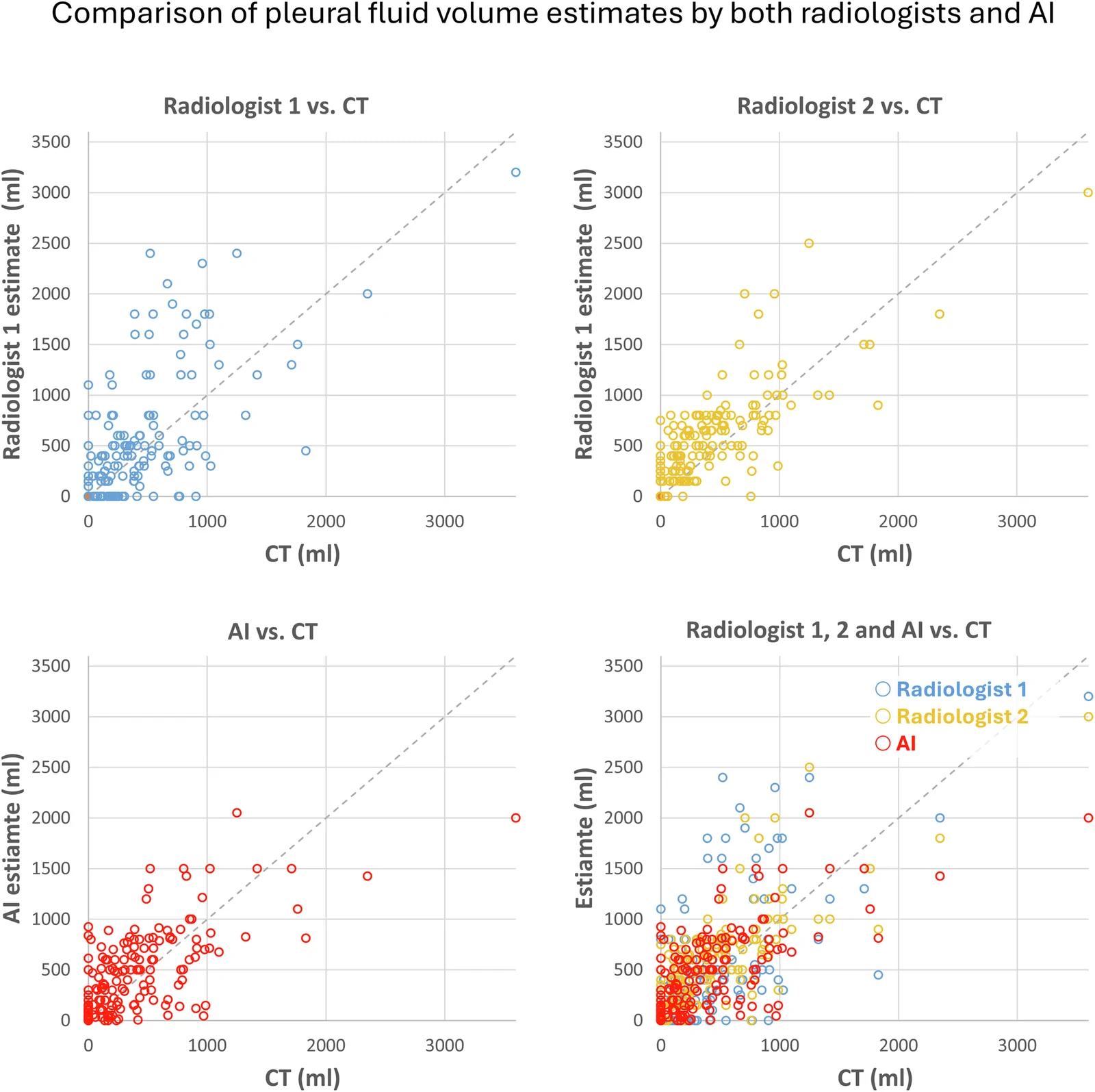
Comparison of pleural fluid volume estimates by both radiologists and AI

The AI analysis was completed successfully for all cases. Using CT as the reference standard, both radiologist and AI-based chest radiograph assessments demonstrated moderate correlation with pleural effusion volume. ICCs were 0.64 (95% CI 0.49–0.77) and 0.74 (95% CI 0. 63–0.83) for radiologist 1 and radiologist 2, respectively, and 0.66 (95% CI 0.56–0.73) for the AI-based estimates (Fig. 3 and Table 1). The mean estimation error was 300 mL (95% CI 252–356 mL) for radiologist 1, 250 mL (95% CI 216–285 mL) for radiologist 2, and 264 mL (95% CI 227–299 mL) for the AI. Median AE was 174 mL for radiologist 1, 191 mL for radiologist 2, and 195 mL for the AI.

**Table 1:** Performance metrics for volume assessment of pleural fluid for both radiologists and AI compared to CT-based measurement

Parameter	R1		R2		AI	
	Value	95% CI	Value	95% CI	Value	95% CI
ICC	0.64	0.49 to 0.77	0.74	0.63 to 0.83	0.66	0.56 to 0.73
Mean error (mL)	300	252 to 356	250	216 to 285	264	227 to 299
Median error (mL)	174	145 to 222	191	150 to 245	195	1550 to 234
Mean bias (mL)	103	45 to 164	146	105 to 186	57	12 to 102
Lower LOA (mL)	-776	-887 to -661	-457	-545 to -366	-651	-770 to -527
Upper LOA (mL)	982	817 to 1131	749	652 to 836	765	661 to 863

*R1* Radiologist 1, *R2* Radiologist 2, *AI* Artificial intelligence, *LOA* Limit of agreement

**Fig. 4:**
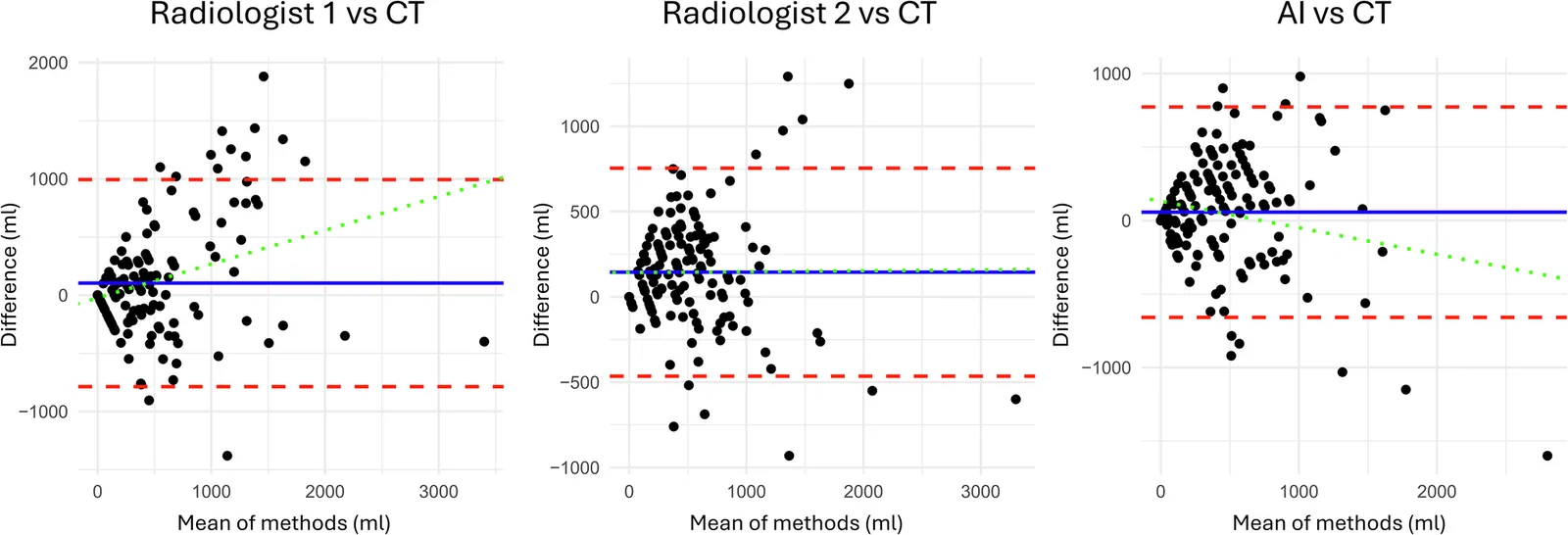
Bland–Altman plot showing mean bias (blue line), limits of agreement (red dashed lines) and proportional bias (green dotted line) for both radiologists and AI in comparison with CT-based measurement of pleural fluid volume

Bland–Altman analysis demonstrated systematic positive bias for both radiologists and the AI algorithm (Fig. 4). Mean bias was +103 mL (95% CI 45–164 mL) for radiologist 1, +146 mL (95% CI 105–186 mL) for radiologist 2, and +57 mL (95% CI 12–102 mL) for AI. Limits of agreement (LOA) ranged from -776 to +982 mL for radiologist 1, -457 to +749 mL for radiologist 2, and -651 to +765 mL for AI-based estimates. The total width of the LOA was therefore 1,758 mL for radiologist 1, 1,206 mL for radiologist 2, and 1,416 mL for the AI (Fig. 4). Mean differences between methods were significant for radiologist 2 versus AI (*p* < 0.001) (Suppl. Table S1 and Suppl. Fig. S1).

Regression analysis of differences in estimates demonstrated significant proportional bias for radiologist 1 (*β* = 0.29, 95% CI 0.17 to 0.41, *p* < 0.001), and for AI (*β* = -0.18, 95% CI -0.30 to -0.06, *p* = 0.005), whereas no proportional bias was observed for radiologist 2 (*β* = 0.00, 95% CI -0.09 to 0.10, *p* = 0.924) (Fig. 4). Calibration plots are shown in Suppl. Fig. S2.

**Fig. 5:**
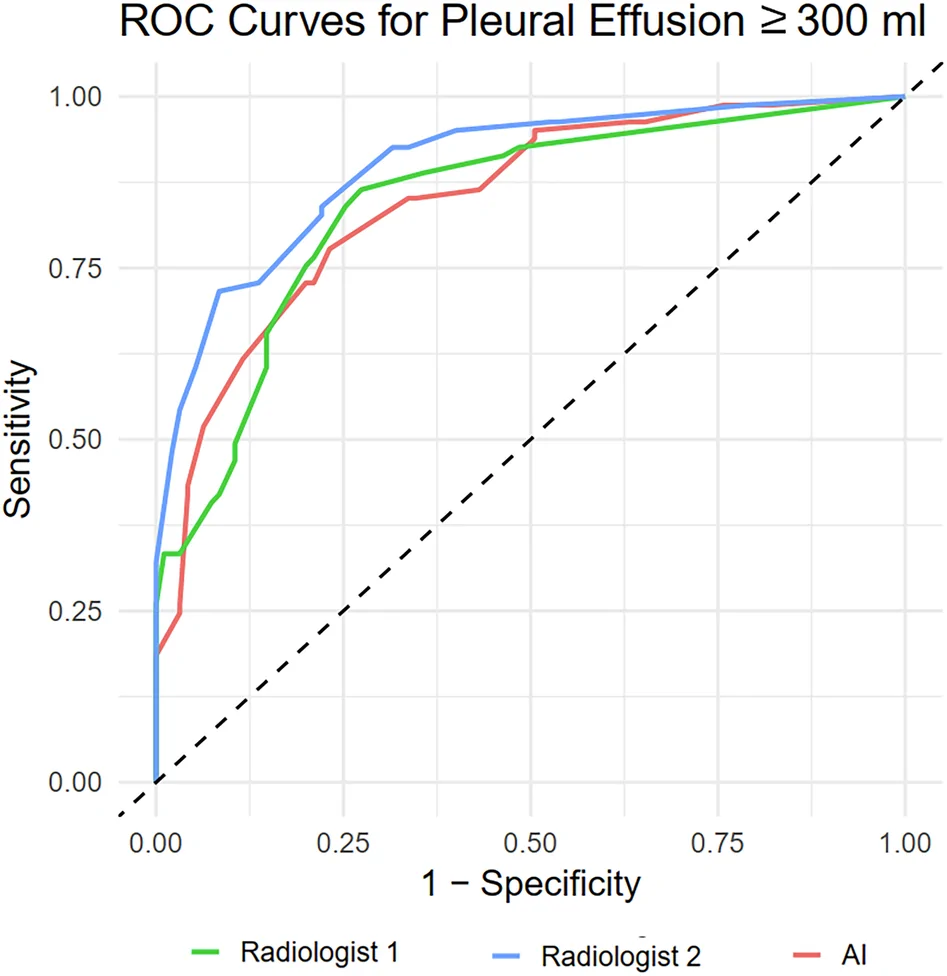
Receiver-operator curve for the detection of clinically significant pleural effusions for both radiologists and AI

The optimal volume threshold using the Youden index for the AI to detect clinically significant pleural effusions (≥ 300 mL) was 380 mL with a sensitivity of 0.78 and a specificity of 0.78 (Fig. 5 and Table 2). The difference in AUC was significant between R1 and R2 (*p* = 0.009), and R2 and AI (*p* = 0.046), while between R1 and AI (*p* = 0.849) it was not, according to the DeLong test.

**Table 2:** Comparison of accuracy for the detection of pleural effusion above ≥ 300 mL for both radiologists and AI

Method	AUC	95% CI	Cutoff (mL)	Sens	Spec	PPV	NPV	Acc
R1	0.84	0.79–0.89	227	0.85	0.76	0.75	0.85	0.79
R2	0.90	0.85-0.94	513	0.81	0.84	0.82	0.85	0.83
AI	0.85	0.79-0.90	380	0.78	0.78	0.76	0.81	0.78

*R1* Radiologist 1, *R2* Radiologist 2, *AUC* Area under the curve, *Sens* Sensitivity, *Spec* Specificity, *PPV* Positive predictive value, *NPV* Negative predictive value, *Acc* Accuracy

Regression analysis showed different predictors of estimation error across methods. For radiologist 1, age (*β* = -6.72 mL per year, *p* = 0.009) and female sex (*β* = 206 mL, *p* = 0.005) were significant predictors of error. For radiologist 2, CT-derived effusion volume (*β* = -0.19, *p* < 0.001) and effusion side (*β* = 121, *p* = 0.003) were significant predictors. For the AI, CT-derived effusion volume (*β* = -0.38, *p* < 0.0001), left side (*β *= 80.2, *p* = 0.040), and patient position (upright *versus* supine *β* = -148, *p* = 0.012) were independently associated with estimation error (Suppl. Table S2).

Confidence was not significantly associated with estimation error for any of the evaluated methods. Radiologist confidence (R1 and R2) showed no significant relationship with error relative to CT (*β* = -8.0 mL, *p* = 0.848; *β* = 10.0 mL, *p* = 0.780, respectively), and AI confidence was likewise not significantly associated with error (*β* = 42.2 mL, *p* = 0.490).

The AI model showed high within-session output consistency, with an ICC of 0.99 (95% CI 0.97–0.99), whereas between-session consistency was lower with an ICC of 0.88 (95% CI 0.81 to 0.96). The repeatability of volumetric measurements was also high at both the intra-observer level (ICC 0.98; 95% CI 0.96–0.99) and the inter-observer level (ICC 0.98; 95% CI 0.96-0.99). In contrast, repeatability for radiologist-based volume assessment was lower, with ICCs of 0.87 (95% CI 0.78–0.95) for radiologist 1 and 0.91 (95% CI 0.85–0.97) for radiologist 2. Inter-observer agreement between the two radiologists was good, with an ICC of 0.88 (95% CI 0.80–0.96).

Outlier analysis identified one statistically significant outlier. A sensitivity analysis of the performance metrics for volume assessment showed comparable results (Suppl. Table S3). Mild increase in proportional bias was noted for radiologist 1 (*β* = 0.41, 95% CI 0.29 to 0.54, *p* < 0.001), while for radiologist 2 (*β* = 0.09, 95% CI -0.01 to 0.20, *p *= 0.092) and AI (*β* = -0.05, 95% CI -0.18 to 0.08, *p* = 0.461) the bias remained minimal.

**Fig. 6:**
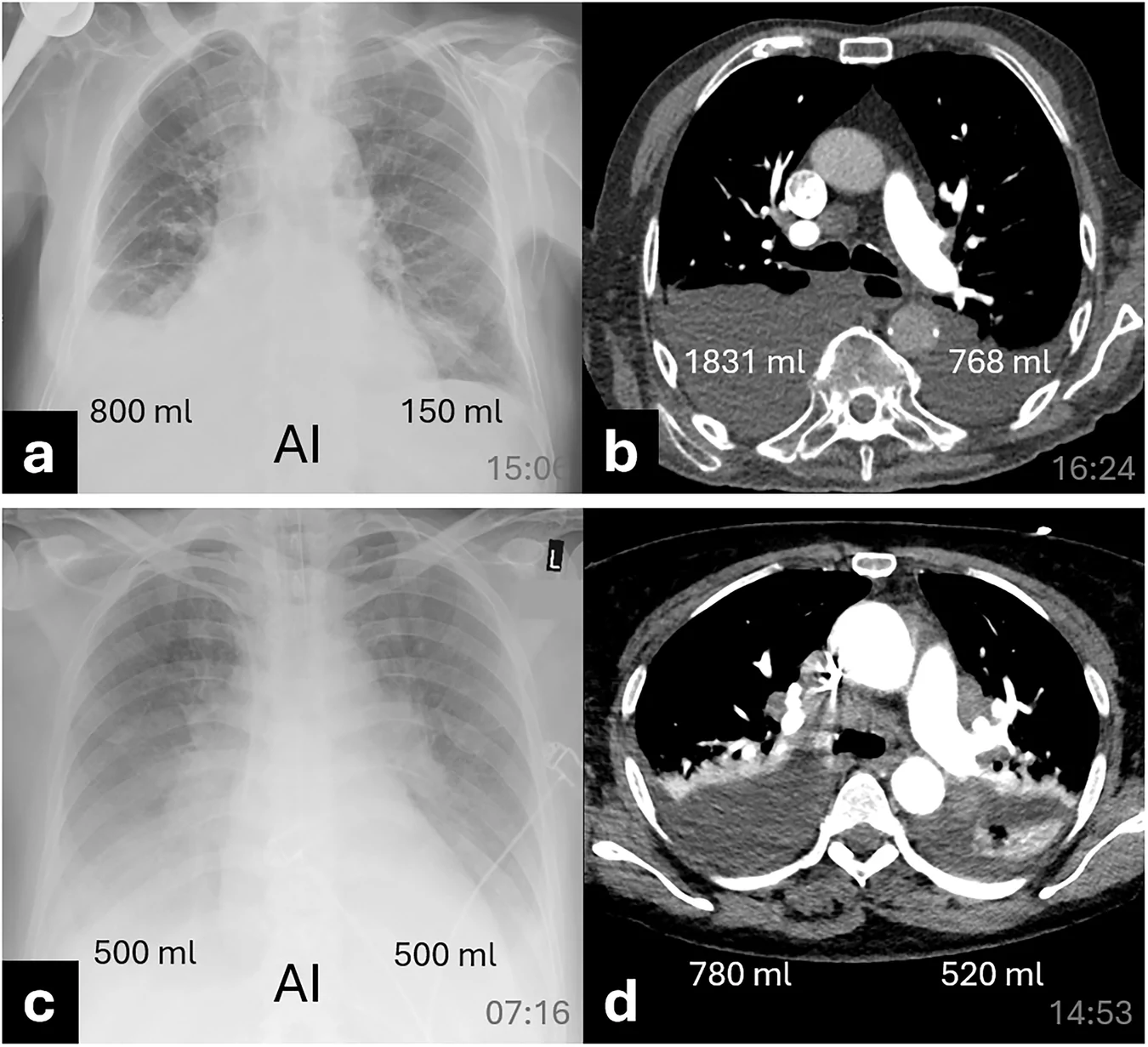
Underestimation of pleural fluid volume on erect chest radiography by AI (**a**) compared with CT volumetry (**b**) in a 93-year-old patient with increased anteroposterior chest diameter. In contrast, accurate estimation of pleural fluid volume on supine chest radiography by AI (**c**) compared with CT volumetry (**d**) in a 52-year-old female patient

Representative cases demonstrating low and high estimation error in pleural fluid volume are shown in Fig. 6.

## Discussion

This study demonstrates that a zero-shot general-purpose large language multimodality model can produce quantitative estimates of pleural effusion volume on chest radiography with accuracy comparable to experienced radiologists, using CT volumetry as the reference standard. Across both radiologists and AI, the observed correlations with CT-derived volumes were moderate, and the magnitude of AE was substantial, highlighting both the inherent limitations of radiographic quantification of pleural fluid and the comparable error of human and AI estimators within this constrained diagnostic context. Supine patient positioning and left-sided pleural effusions—well-recognized confounders in pleural effusion detection— pose greater challenges to AI than to the radiologists.

A novel finding of this analysis is that the AI-based estimates showed agreement with CT comparable to that achieved by the two board-certified radiologists [9]. ICC correlation coefficients ranged between 0.64 and 0.74 across readers and the AI model, while mean AEs were approximately 250–300 mL. These results suggest that when provided with a structured prompt, a general multimodal AI model can extract radiographic cues relevant to pleural effusion quantification at a level comparable to human interpretation [2].

The observation that both radiologists and the AI demonstrated substantial systematic positive bias and error reflects intrinsic limitations of chest radiography rather than reader-specific errors. Radiographic signs such as costophrenic angle blunting, meniscus formation, and decreased transparency of a hemithorax represent indirect geometric manifestations of pleural fluid that inherently constrain accurate volumetric estimation [2, 10]. Wide LOA, exceeding 1 L, confirms that chest radiography cannot reliably quantify pleural fluid volume, regardless of whether the interpretation is performed by radiologists or AI [11].

Previously, assessments of pleural effusion on chest radiography have been largely semiquantitative. Early work suggested that the appearance of the meniscus sign on posteroanterior chest plain film radiographs corresponds to approximately 200 mL of pleural fluid, a value that has subsequently been regarded as the minimum threshold for radiographic detectability [2]. A later study (2015) refined these estimates, reporting that a meniscus sign confined below the hemidiaphragm corresponded to roughly 100 mL, whereas extension above the hemidiaphragm was associated with approximately 250 mL [10]. To date, a fully quantitative estimation of pleural effusion volume from chest radiographs has not been systematically explored; the present study represents an initial exploration of this area. Although such estimates are inherently approximate, they may still support clinical decision-making. Small pleural effusions are frequently managed conservatively with clinical and imaging follow-up, whereas larger effusions, particularly those exceeding approximately 300–500 mL, are more likely to prompt further evaluation with ultrasound or CT and may necessitate therapeutic interventions such as thoracentesis, pleural drainage, or intensification of diuretic therapy [8, 11]. Continuous volumetric estimation may therefore provide meaningful information beyond purely qualitative classification, particularly in settings where cross-sectional imaging is not immediately available.

The use of general-purpose multimodal models for quantitative imaging tasks remains an emerging area and should be interpreted with caution. Most prior AI studies have focused on binary detection of pleural effusion, whereas quantitative volume estimation represents a more complex task. Specialized convolutional neural network architectures, particularly EfficientNet-based models, have achieved reported accuracies of approximately 93%–95% for identifying the presence of pleural effusion, substantially outperforming general multimodal systems such as ChatGPT, which demonstrated detection rates around 70–75% in exploratory analyses [6, 7, 12]. A pilot study by Lacaita et al reported that ChatGPT-4o correctly identified 70% (21/30) of obvious pleural effusions but did not evaluate quantitative aspects such as effusion volume or specificity [6]. In contrast, Huang et al reported 93% accuracy using an EfficientNet-B0 model; however, their dataset appears to have been preselected based on radiologist labels (normal *versus* pleural effusion), potentially introducing spectrum bias relative to real clinical workflows [12].

Performance of commercially available AI systems shows somewhat lower sensitivity but remains clinically meaningful: reported detection rates include sensitivity of 0.74 (AUC 0.86) compared with 0.88 for radiologist reports based on consensus ground truth, and sensitivity around 60% (AUC 0.80) compared with 67% for radiology residents when senior radiologists defined the reference standard [13, 14]. A cross-vendor evaluation from Germany comparing systems from Gleamer and AZmed with radiologist interpretation reported sensitivities between 0.71 and 0.85, although a clearly defined ground truth was lacking [5]. Sensitivity was slightly higher in upright radiographs (approximately 69%–91%) than in supine examinations (63%–70%).

Studies applying ChatGPT-4.0 to labeled datasets such as the NIH ChestX-ray database similarly reported sensitivities of approximately 0.75 for pleural effusion detection [7]. Conversely, highly optimized deep learning algorithms trained specifically for fluidothorax detection have achieved very high performance metrics, including AUC values up to 0.97 with sensitivity around 95%, although these studies typically rely on radiologist consensus as the reference standard rather than on a superior method such as CT as the gold standard [15].

Attempts to extend AI beyond binary detection toward quantitative assessment have been more limited; one proprietary deep learning model, trained on the MIMIC-CXR dataset, classified pleural effusion volume into four categories derived from report labels, achieving an overall accuracy of 73% [16]. Overall, most studies focus on binary presence/absence classification and often rely on radiologist annotations rather than independent ground truth, but the available evidence suggests that AI assistance can increase detection sensitivity by approximately 9% compared with unaided interpretation [4].

The regression analyses revealed several factors associated with estimation error. For the AI model, CT-derived effusion volume, effusion laterality, and patient position were significant predictors. Upright radiographs were associated with lower estimation error compared with supine acquisitions. This observation is consistent with established radiographic principles [2, 17]. In the supine position, the resulting radiographic appearance often resembles diffuse opacity rather than discrete fluid layering, making volumetric inference more challenging [5, 18]. Assessment of left-sided pleural effusions may be more challenging due to the superimposition of the heart silhouette.

Interestingly, confidence scores were not significantly associated with estimation accuracy for either radiologists or the AI model. This lack of association likely reflects the inherent difficulty of pleural effusion assessment on chest radiography, which requires nuanced visual interpretation and is subject to multiple confounding factors. In contrast, tasks that rely primarily on factual knowledge retrieval or structured reasoning may demonstrate better calibration between confidence and accuracy [19–21].

Several limitations of this study should be acknowledged. First, the study was conducted retrospectively at a single tertiary institution and included a relatively small sample size, which may limit the generalizability of the findings to other clinical settings or patient populations. The cohort consisted exclusively of adult patients, and therefore, the results cannot be directly extrapolated to pediatric populations. Second, the study cohort comprised patients with pleural effusion of heterogeneous etiologies, radiographs in different patient positions; this heterogeneity reflects real-world clinical practice but may also represent a source of variability in estimation accuracy. Third, although generally short, the interval between chest radiography and CT could allow minor changes in pleural fluid volume between the examinations. Fourth, CT-derived volumetry was used as the reference standard; however, no comparison with actual drained fluid volume was performed, and therefore, a true physical reference standard was not available. Fifth, the selection strategy resulted in enrichment of positive cases and introduced spectrum bias. Sixth, repeatability may be limited by the use of a non-locked commercial AI model that is subject to iterative updates, which may affect the consistency of outputs over time. Finally, the evaluation of AI was limited to a single general-purpose multimodal model using a specific zero-shot prompting strategy. Future studies should include larger, multicenter cohorts and evaluate the performance of optimized AI models using cross-sectional imaging as the reference standard.

In conclusion, quantitative estimation of pleural effusion volume on chest radiography shows only moderate agreement with CT-derived volumetry, regardless of whether interpretation is performed by experienced radiologists or by a specific general-purpose commercial multimodal AI model. Zero-shot prompting of the AI system produced volumetric estimates with accuracy comparable to those of radiologists. Supine radiographs and left-sided pleural effusions were associated with greater estimation error for the AI model. The wide LOA observed across all evaluators in this retrospective cohort confirms that chest radiography provides only a coarse approximation of pleural fluid volume, irrespective of the interpreter. While it may support initial triage of effusion burden, accurate quantification requires cross-sectional imaging such as ultrasound or CT.

## Supplementary information

**Additional file 1: Fig. S1**. Pairwise comparisons of mean differences of pleural fluid volume among radiologist 1 (R1), radiologist 2 (R2), and AI. **Fig. S2**. Calibration plots for radiologist 1 (R1), radiologist 2 (R2), and AI. **Suppl. Table 1**. Pairwise comparisons of mean differences of pleural fluid volume among radiologist 1 (R1), radiologist 2 (R2), and AI. **Suppl. Table 2**. Predictors of estimation error of pleural fluid volume for Radiologist 1, Radiologist 2, and AI. **Suppl. Table 3**. Performance metrics for volume assessment of pleural fluid for both radiologists and AI compared to CT-based measurement after exclusion of an outlier.

## Data Availability

The datasets generated and analyzed during the current study are not publicly available due to institutional and data protection regulations, but are available from the corresponding author on reasonable request.

## Abbreviations

AE: Absolute error
AI: Artificial intelligence
AUC: Area under the curve
CI: Confidence interval
CT: Computed tomography
ICC: Intraclass correlation coefficient
IQR: Interquartile range
LOA: Limits of agreement
Q–Q plot: Quantile–quantile plot
*β*: Regression coefficient
