# Supplementary material for: Quantitative estimation of pleural effusion volume on chest radiography: comparison of radiologists and a general-purpose multimodal AI model using CT as the reference standard
Source: Eur Radiol Exp. 2026 Sep 10;10:126. doi: 10.1186/s41747-026-00790-3 (PMC13562479; doi:10.1186/s41747-026-00790-3)
Supplement: Supplementary file 1 [file 41747_2026_790_MOESM1_ESM.pdf]

Quantitative estimation of pleural effusion volume on chest radiography: comparison of radiologists and a general-purpose multimodal AI model using CT as the reference standard

ELECTRONIC SUPPLEMENTARY MATERIAL

No.	STARD-AI Item	Reported on page / location
**Title or abstract**		
1	Identification as a study reporting AI-centred diagnostic accuracy and reporting at least one measure of accuracy within title or abstract	Title page; Abstract (Background, Methods, Results, Conclusions). Title contains "general-purpose multimodal AI model"; Abstract reports ICC, mean absolute error, AUC.
**Abstract**		
2	Structured summary of study design, methods, results and conclusions	Abstract (page 2): structured into Background, Methods, Results, Conclusions sections.
**Introduction**		
3	Scientific and clinical background, including the intended use of the index test, whether it is novel or an established index test, and its integration into an existing or new workflow	Background section, paragraphs 1–2: clinical context of pleural effusion, limitations of chest radiography, role of AI in radiograph analysis, novelty of quantitative volume estimation.
4	Study objectives and hypotheses	Background section, last paragraph: study aim and hypothesis explicitly stated ("We hypothesized that AI would achieve accuracy comparable to radiologists, while both approaches would show only moderate agreement with CT").
**Methods — Study design**		
5	Whether data collection was planned before the index test and reference standard were performed (prospective study) or after (retrospective study)	Abstract Methods and Methods, opening paragraph: "retrospective observational study".
**Methods — Ethics**		
6	Formal approval from an ethics committee. If not required, justify why	Methods, opening paragraph: Ethics Committee approval, waiver of informed consent. Declarations: EK-17/23, 11/2/2026.
**Methods — Participants**		
7	Eligibility criteria: listing separate inclusion and exclusion criteria in the order that they are applied at both participant level and data level	Methods, "Study Population and Inclusion Criteria": bullet list of inclusion and exclusion criteria.
8	On what basis potentially eligible participants were identified	Methods, Study Population: identified through retrospective search of the institutional radiology information system for CT reports mentioning pleural effusion and synonyms.
9	Where and when potentially eligible participants were identified (setting, location, and dates)	Methods (Motol and Homolka University Hospital, Prague, Czech Republic; January 1 – December 31, 2025); Results, opening paragraph.
10	Whether participants formed a consecutive, random, or convenience series	Methods, Study Population: "Consecutive patients were identified".
**Methods — Dataset**		
11	Source of the data and whether it has been routinely collected, specifically collected for the purpose of the study or acquired from an open-source repository	Methods, Study Population: routinely collected clinical data from the institutional radiology information system (retrospective).
12	Who undertook the annotations for the dataset and how (within the same clinical context or in a post-hoc fashion)	Methods, CT Evaluation: radiology resident performed initial segmentation; second board-certified radiologist re-segmented 20 randomly selected patients. Methods, Chest Radiograph Evaluation: two board-certified radiologists (R1: 19 years experience; R2: 9 years experience) annotated radiographs.
13	Devices (manufacturer, model) that were used to capture data; software (with version number) used to engineer the index test	Methods, CT Evaluation: four scanners (Aquilion ONE, PRIZM, PRIME, Canon Medical Systems; Somatom Force, Siemens Healthineers); Syngo Via workstation. Methods, AI section: ChatGPT version 5.2 (OpenAI), accessed via Google Chrome v.144.

No.	STARD-AI Item	Reported on page / location
14	Data acquisition protocols and details of data preprocessing in sufficient detail to allow replication	Methods, CT Evaluation: 3 mm slice thickness uniform across scanners; manual segmentation by contouring with extrapolation between slices. Methods, AI section: Windows screen capture tool, default ChatGPT settings (no temperature adjustment), full prompt reproduced verbatim.
**Methods — Test methods**		
15a	Index test, in sufficient detail to allow replication	Methods, AI-based radiograph evaluation: full structured prompt reproduced verbatim, including specific instructions for output format and confidence rating.
15b	How the index test was developed, including any training, validation, testing and external evaluation	Methods, AI section: zero-shot prompting strategy (no model training); model selected after qualitative pre-screening of five LLMs (ChatGPT 5.2, Grok 4.1, Microsoft Copilot, Claude Sonnet 4.5, Gemini 3 Flash). Tested on 88 patients (176 hemithoraces).
15c	Definition of and rationale for test positivity cut-offs or result categories of the index test, distinguishing pre-specified from exploratory	Methods, Statistical analysis: 300 ml threshold for "clinically significant" effusion (pre-specified). ROC analysis labelled exploratory; cut-offs derived in the same enriched sample.
15d	The specified end user of the index test and the level of expertise required of users	Methods, AI section: web interface accessed via standard browser; default settings used; intended for use without specific technical expertise.
16a	Reference standard, in sufficient detail to allow replication	Methods, CT Evaluation: full description of CT scanners, slice thickness, manual segmentation protocol with extrapolation, software used (Syngo Via).
16b	Rationale for choosing the reference standard (if alternatives exist)	Methods, CT Evaluation: CT volumetry chosen as reference standard. Limitations 4: thoracentesis-based volume not used (acknowledged as limitation).
16c	Definition of and rationale for test positivity cut-offs or result categories of the reference standard, distinguishing pre-specified from exploratory	Methods: pleural effusion ≥20 ml on CT used as pre-specified inclusion threshold (rationale: minimum reliable detection volume); ≥300 ml threshold for "clinically significant" effusion (pre-specified).
17a	Whether clinical information and reference standard results were available to the performers or readers of the index test	Methods, Chest Radiograph Evaluation: radiologists "blinded to CT images and measurements and between each other".
17b	Whether clinical information and index test results were available to the assessors of the reference standard	Methods, CT Evaluation: CT segmentation performed first, before radiograph readings; segmenters were not aware of subsequent index test results.
**Methods — Analysis**		
18	Methods for estimating or comparing measures of diagnostic accuracy	Methods, Statistical analysis: ICC (two-way mixed-effects, absolute agreement), absolute error, Bland–Altman analysis, ROC with Youden index, DeLong test for AUC comparison, Friedman test with Wilcoxon signed-rank post-hoc, exploratory linear regression.
19	How indeterminate index test or reference standard results were handled	Methods, AI section: "AI analysis was successful in all cases" (no indeterminate results requiring handling).
20	How missing data on the index test and reference standard were handled	Methods, exclusion criteria: "Unavailable data" listed as exclusion criterion. No imputation performed.
21	Any analyses of variability in diagnostic accuracy, distinguishing pre-specified from exploratory	Methods, Statistical analysis: subgroup analyses (regression with predictors of error) labelled "Exploratory linear regression models". Sensitivity analysis (outlier exclusion via Grubbs test) explicitly labelled secondary.

No.	STARD-AI Item	Reported on page / location
22	Intended sample size and how it was determined	Methods, Statistical analysis: paired-design power calculation (effect = 100 ml, SD of paired differences = 200 ml, α = 0.05, power = 80%); minimum required = 32 paired observations.
23	Details of any performance error analysis, and algorithmic bias and fairness assessments if undertaken	Methods, Statistical analysis (regression analysis of error predictors). Discussion: errors associated with patient position, side, age, sex, etiology of pleural effusion. Algorithmic fairness not separately assessed; partly addressed in Limitations.
**Results — Participants and dataset**		
24	Flow of participants, using a diagram	Figure 1 (study flowchart) cited in Results, opening paragraph.
25	Baseline demographic, clinical and technical characteristics of training, validation and test set	Results, demographics paragraph: age, sex, BMI, time interval between radiograph and CT, patient position distribution.
26a	Distribution of severity of disease in those with the target condition	Results: median pleural fluid volume 389 ml (IQR 188–703 ml); etiology distribution; Figure 2 (volume distribution by side).
26b	Distribution of alternative diagnoses in those without the target condition	Not applicable. Study cohort restricted to patients with CT-confirmed pleural effusion ≥20 ml; no "without target condition" subgroup.
27	Time interval and any clinical interventions between index test and reference standard	Methods (same calendar day inclusion criterion); Results: "median time interval between chest radiography and CT was 3.0 hours (IQR 1.50–5.19 hours)". Eligibility excluded "pleural effusion drainage between chest X-ray and CT".
28	Whether the datasets represent the distribution of the target condition that one would expect from the intended use population	Methods, Study Population: explicit statement of enrichment of positive cases and spectrum bias. Limitations 5: spectrum bias acknowledged.
29	For external evaluation on an independent dataset, an assessment of how this differs from the training, validation and test sets	Not applicable. AI was used in zero-shot mode without training or fine-tuning; no separate independent dataset for external evaluation. Single-cohort retrospective study at one institution.
**Results — Test results**		
30	Cross tabulation of the index test results (or their distribution) by the results of the reference standard	Table 2 (sensitivity, specificity, PPV, NPV, accuracy at ≥300 ml threshold). Figure 3 (correlation plots index test vs. reference). Figure 4 (Bland–Altman analysis).
31	Estimates of diagnostic accuracy and their precision (such as 95% confidence intervals)	Table 1 (ICC, mean error, median error, mean bias, LOA — all with 95% CI). Table 2 (AUC with 95% CI). Suppl. Tables 1, 2, 3.
32	Any adverse events from performing the index test or the reference standard	Not applicable. Both index test (radiograph interpretation by AI) and reference standard (CT volumetry) were retrospective imaging analyses with no patient-facing intervention or risk.
**Discussion**		
33	Study limitations, including sources of potential bias, statistical uncertainty, and generalisability	Limitations section: 7 items listed (single-centre retrospective design, cohort heterogeneity, time interval between modalities, no thoracentesis comparison, spectrum bias, non-locked AI model with iterative updates, single-model evaluation).
34	Implications for practice, including the intended use and clinical role of the index test	Conclusions: "may support initial triage of effusion burden; accurate quantification requires cross-sectional imaging such as ultrasound or CT". Discussion final paragraph: clinical positioning.
35	Ethical considerations and adherence to ethical standards associated with the use of the index test and issues of fairness	Methods, Ethics; Declarations: ethics approval, waiver of informed consent due to retrospective use of anonymized data. Algorithmic fairness —

No.	STARD-AI Item	Reported on page / location
		assessed via regression analysis of demographic predictors of error.
**Other information**		
**36**	Registration number and name of registry	Declarations: ethics approval reference number EK-17/23, 11/2/2026, Ethics Committee of Motol and Homolka University Hospital. Retrospective observational study; no separate trial registration applicable.
**37**	Where the full study protocol can be accessed	Not applicable. Retrospective observational study without separately published protocol.
**38**	Sources of funding and other support; role of funders	Declarations: Ministry of Health Czech Republic (MH CZ-DRO, Motol University Hospital, 00064203); Charles University (Cooperatio, Medical Diagnostics and Basic Medical Sciences). Institutional funding without role in design, analysis, or reporting.
**39**	Commercial interests, if applicable	Declarations: "The authors declare that they have no competing interests."
**40a**	Availability of datasets and code; detailing any restrictions on their reuse and repurposing	Declarations: "The datasets generated and analyzed during the current study are not publicly available due to institutional and data protection regulations but are available from the corresponding author on reasonable request." AI prompt reproduced verbatim in Methods to allow replication.
**40b**	Whether outputs are stored, auditable and available for evaluation, if necessary	AI outputs (volume estimates and confidence scores) and CT volumetric segmentations stored in institutional research database; available from corresponding author on reasonable request, subject to institutional and data protection regulations.

Supplementary Figures

Suppl. Figure S1

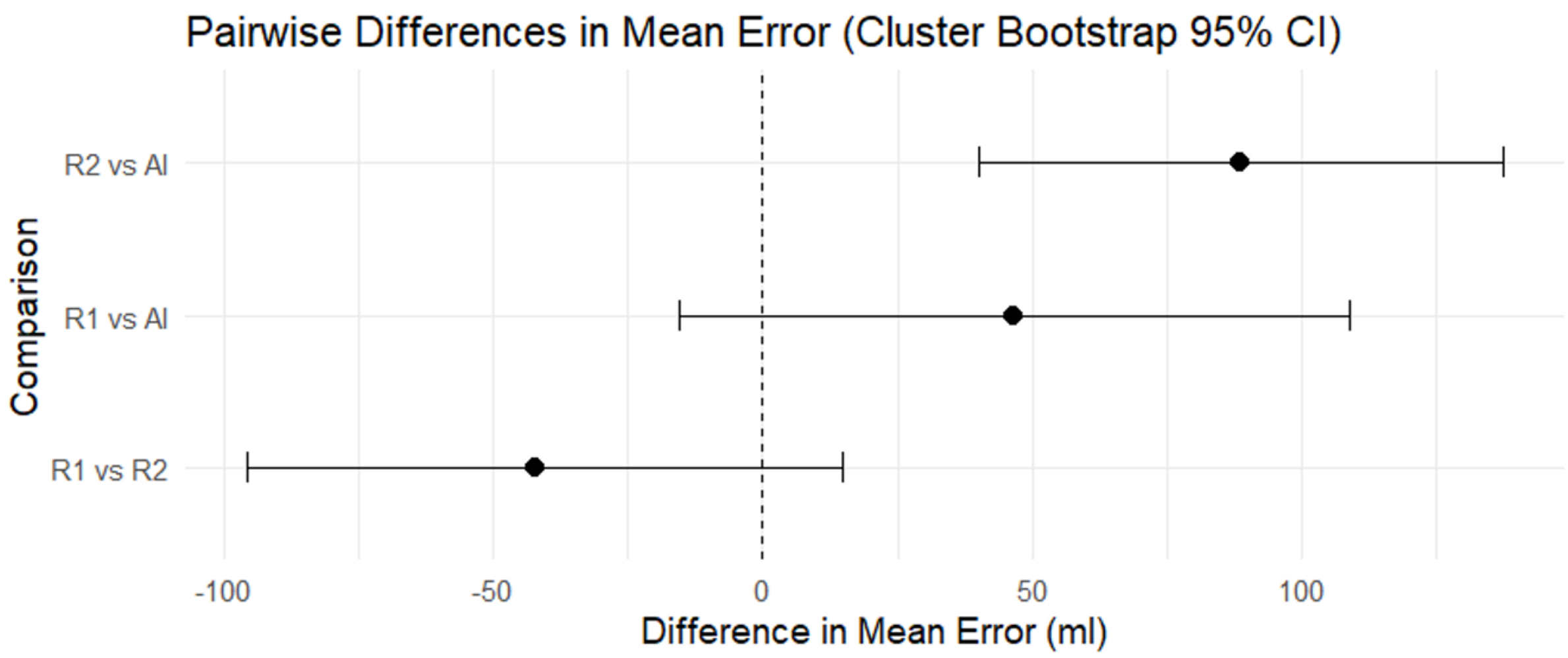

Fig. S1. Pairwise comparisons of mean differences of pleural fluid volume among radiologist 1 (R1), radiologist 2 (R2), and AI.

Suppl. Figure S2

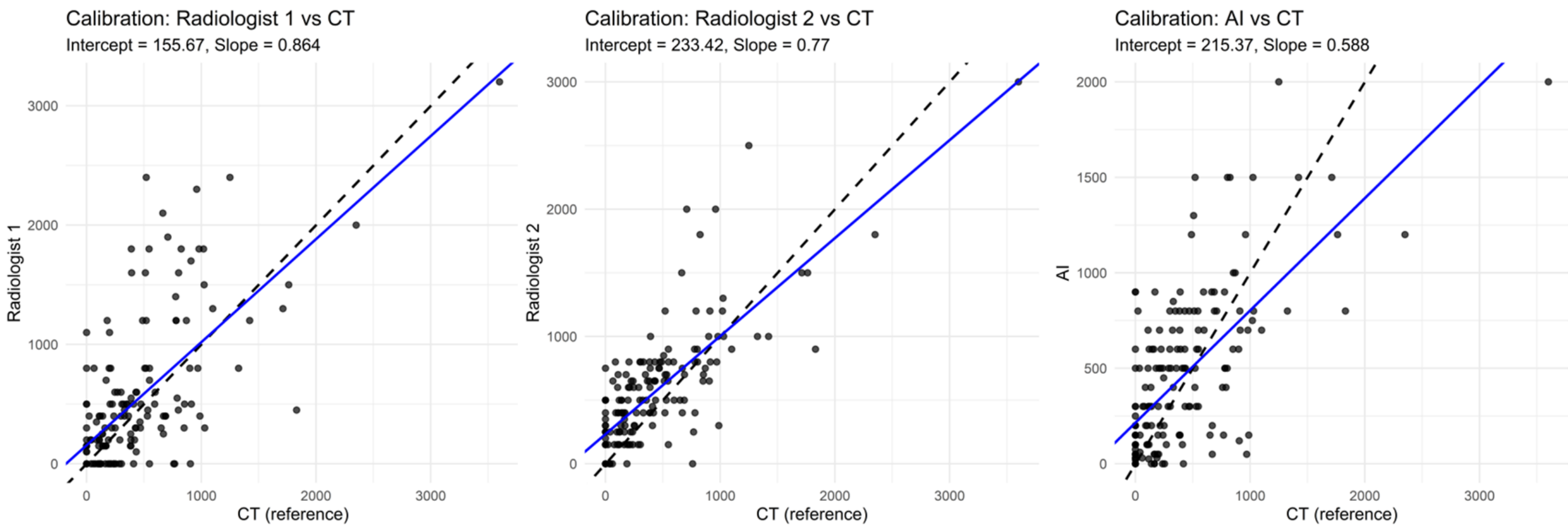

Fig. S2. Calibration plots for radiologist 1 (R1), radiologist 2 (R2), and AI.

Supplementary Tables

Suppl. Table 1. Pairwise comparisons of mean differences of pleural fluid volume among radiologist 1 (R1), radiologist 2 (R2), and AI.

Comparison	Mean difference (ml)	95% CI	p-value
R1 vs R2	-42	-95 to 15	0.127
R1 vs AI	46	-16 to 109	0.150
R2 vs AI	89	40 to 137	<0.0001

R1, radiologist 1; R2, radiologist 2; AI, artificial intelligence

Suppl. Table 2. Predictors of estimation error of pleural fluid volume for Radiologist 1, Radiologist 2, and AI.

	Radiologist 1			Radiologist 2			AI		
**Predictor**	**β (ml)**	**95% CI**	**p-value**	**β (ml)**	**95% CI**	**p-value**	**β (ml)**	**95% CI**	**p-value**
**CT volume (ml)**	-0.07	-0.21 to 0.06	0.29	-0.19	-0.28 to -0.1	<0.0001	-0.38	-0.47 to -0.29	<0.0001
**Age**	-6.72	-11.63 to -1.81	0.0088	-2.56	-5.74 to 0.62	0.12	-1.18	-4.69 to 2.33	0.51
**Sex (female vs male)**	205.76	64.79 to 346.72	0.0054	84.73	-6.68 to 176.15	0.073	64.32	-36.6 to 165.24	0.22
**BMI**	2.04	-10.11 to 14.19	0.74	3.73	-4.15 to 11.61	0.36	-1.49	-10.19 to 7.2	0.73
**Side (right vs left)**	110.10	-6.36 to 226.55	0.067	121.73	43.07 to 200.39	0.0033	80.19	4.96 to 155.42	0.040
**Position sitting vs supine**	74.90	-116.09 to 265.89	0.44	61.96	-61.89 to 185.82	0.33	73.01	-63.71 to 209.73	0.30
**Position standing vs supine**	-73.64	-230.13 to 82.85	0.36	46.32	-55.16 to 147.8	0.37	-147.75	-259.78 to -35.72	0.012

β, regression coefficient; CI, confidence interval; BMI, body mass index.

Suppl. Table 3. Performance metrics for volume assessment of pleural fluid for both radiologists and AI compared to CT-based measurement after exclusion of an outlier.

Parameter	R1		R2		AI	
	Value	*95%CI*	Value	*95%CI*	Value	*95%CI*
**ICC**	0.57	0.46 to 0.68	0.69	0.61 to 0.76	0.62	0.52 to 0.71
**Mean error (ml)**	300	247 to 359	247	213 to 282	257	224 to 290
**Median error (ml)**	172	140 to 222	187	150 to 228	195	157 to 234
**Mean bias (ml)**	105	45 to 166	149	112 to 192	66	23 to 107
**Lower LOA (ml)**	-772	-878 to -659	-445	-529 to -350	-602	-694 to -499
**Upper LOA (ml)**	982	816 to 1138	744	648 to 830	734	650.50 to 812

R1, radiologist 1; R2, radiologist 2; AI, artificial intelligence; LOA, limits of agreement
